# Preparation of an Omental Pedicle Flap in Cats and Dogs Through a Lateral Approach Laparotomy—A Cadaver Study

**DOI:** 10.3390/ani16091341

**Published:** 2026-04-28

**Authors:** Tom Adrian Ablassmaier, Stefana Maria Cristina Muresan, Liviu Ioan Oana, Adrian Todor, Lucia Victoria Bel

**Affiliations:** 1 Department of Surgery, Anesthesiology and Intensive Care, Faculty of Veterinary Medicine, University of Agricultural Sciences and Veterinary Medicine Cluj-Napoca, 400372 Cluj-Napoca, Romania; tom-adrian.ablassmaier@usamvcluj.ro (T.A.A.); oanaliviu2008@yahoo.com (L.I.O.); lucia.bel@usamvcluj.ro (L.V.B.); 2 Department of Pediatric Orthopedics and Traumatology, Faculty of Medicine, “Iuliu Hațieganu” University of Medicine and Pharmacy, 8 Victor Babeș Street, 400012 Cluj-Napoca, Romania; atodor81@gmail.com

**Keywords:** greater omentum, omental pedicle flap, lateral approach laparotomy, extra-abdominal surgery, cadaver study

## Abstract

The use of an omental pedicle flap as an adjuvant treatment has gained popularity in veterinary medicine due to its versatility. This technique has been successfully applied in both intra-abdominal and extra-abdominal surgeries in human as well as veterinary medicine. In most intra-abdominal procedures, the greater omentum can be easily accessed through a midline laparotomy and directed to the area of interest. However, dogs and cats are typically positioned in lateral recumbency for extra-abdominal surgeries involving the thorax or limbs. In such cases, a lateral approach to the greater omentum could minimize the risk of infection or trauma associated with patient repositioning. This cadaver study evaluated the feasibility of accessing the greater omentum through a lateral approach laparotomy in both left and right lateral recumbency. A total of 22 cadavers (13 cats and 9 dogs) were included. Dorsal extension of the greater omentum was successfully performed in all specimens without major structural damage. Further elongation using an inverted L-shaped pedicle flap was achieved without rupture in 17 of 22 cadavers (77.3%). The results demonstrate that lateral approach laparotomy provides reliable access to the greater omentum and allows successful mobilization of the omentum for potential extra-abdominal applications, with no significant differences observed between left and right lateral approaches. This technique may represent a useful alternative in clinical situations where repositioning the patient is not feasible or carries increased surgical risk.

## Simple Summary

The greater omentum is a fatty tissue in the abdomen that supports healing and protects nearby organs. It is widely used in both veterinary and human surgery. Usually, it is accessed through a central abdominal incision. However, in some situations, this approach may increase surgical and anesthetic risks, especially when patients need to be repositioned. This study explores a lateral surgical approach as a possible safer alternative. Thirteen cats and nine dogs were used to evaluate whether the omentum can be safely accessed and extended using anatomical landmarks such as nearby organs and blood vessels. In all cases, the omentum could be successfully exposed, and one extension technique was consistently safe. A second technique allowed further extension but led to rupture in a small number of cases. These findings suggest that a lateral approach may provide a useful and safer option for surgeons when standard positioning is not possible, potentially reducing surgical risks and improving patient outcomes.

## 1. Introduction

In recent years, the greater omentum has garnered significant attention from surgeons in both human and veterinary medicine. However, its potential in soft tissue surgery remains underutilized by many practitioners, and data on its use in orthopedic applications are still limited [1]. The increasing interest in soft tissue applications originates from the omentum’s remarkable properties, including its ability to promote angiogenesis, facilitate drainage, and provide immunologic and adhesive support [1,2,3]. It is rich in vascular and lymphatic supply and is covered by a mesothelial membrane [1,2].

In human and veterinary medicine, these characteristics make the greater omentum a valuable option for reconstructing the chest or abdominal wall, managing chronic non-healing wounds of both soft and hard tissues outside the abdominal cavity, and enveloping organs within the abdominal cavity after invasive procedures [1,4,5,6,7,8].

In veterinary medicine, the use of an omental pedicle flap has only recently gained attention from surgeons. In contrast, research in human medicine has progressed since the early 20th century due to numerous scientists investigating the role of the greater omentum, its anatomy and its potential applications in surgical procedures [9,10].

Early investigations into the therapeutic use of the greater omentum were conducted in human medicine. Among the pioneers was Goldsmith, who systematically described the surgical potential of the greater omentum in his comprehensive work *The Omentum* [8].

In addition, Chiricuță and Goldstein published articles describing the use of the greater omentum during the repair of vesicovaginal fistulas [11,12].

In veterinary medicine, omental pedicle techniques were introduced later. However, the anatomy of the canine omentum had already been described in detail in the early 20th century [9]. Usually, the greater omentum, specifically its largest component—the bursal portion—floats over the intestinal loops. Its superficial leaf is attached along the entire length of the greater curvature of the stomach, extending from the fundus to the pylorus and continuing caudally along the proximal duodenum. From there, the superficial leaf courses dorsally and transitions into the deep leaf, which attaches to the dorsal abdominal wall and extends toward the left lobe of the pancreas. On the left side, the splenic artery separates the deep leaf of the bursal portion from the splenic portion of the greater omentum [9,13].

Within the abdominal cavity, the omentum can often be used without needing a pedicle flap, while applications outside the abdominal cavity necessitate surgical skills and a comprehensive understanding of the omentum’s vascularization and physiology.

Creating a viable omental pedicle flap involves separating the deep leaf of the bursal portion from its attachments or, depending on the technique, partially mobilizing both the superficial and deep leaves [2,14]. Surgeons may also extend this process further by creating an elongated inverted L-shaped omental pedicle flap that begins caudal to the gastrosplenic ligament and then continues caudally [2].

In 1993 Ross & Pardo described a technique for lengthening the greater omentum by creating a pedicle omental flap. Their technique involved the dissection of the dorsal leaf of the bursal portion, followed by further elongation of the greater omentum by cutting the ventral leaf partially in order to create an L-shaped flap. This is done by making an incision on the left side of the superficial layer of the bursal portion near the caudal end of the spleen, perpendicular to the full length of the elongated bursal portion. This perpendicular incision is made up to the middle of the bursal portion, and then, moving caudally, the bursal portion is cut almost in half until the caudal border is reached, leaving some vessels to ensure the vascular supply of the elongated L-shaped pedicle flap [2].

A novel approach was introduced by Doom et al. (2016), who investigated a technique to create an omental pedicle flap based on their prior morphological and vascular studies [13,14,15].

This technique consisted of preparing the flap from the right side, medially to the pylorus, by cutting a few centimeters into the deep and superficial leaves of the greater omentum, separating it from its connections to the stomach. This was followed by an incision moving caudally, cutting approximately 2/3 of the length of the greater omentum and then moving medially again, also creating an L-shaped flap [14].

Traditionally, the greater omentum is accessed via a midline celiotomy with the patient in ventro-dorsal recumbency. This poses a challenge if the primary surgery targets another area of the body, as a midline celiotomy requires repositioning the patient. Turning the patient during active surgery can lead to complications, both surgical and anesthetic, as the surgical field may become contaminated.

Therefore, the aim of this cadaver study was to evaluate whether a lateral abdominal approach in latero-lateral recumbency allows reliable exposure and preparation of the greater omentum, including dorsal extension and inverted L-shaped elongation, while eliminating the need for patient repositioning. To date, the feasibility of preparing an omental pedicle flap via a lateral abdominal approach has not been systematically evaluated. Therefore, this study aims to provide a practical guide for practitioners who wish to apply this technique in clinical cases. Avoiding repositioning may reduce mechanical risks, such as inadvertent stretching or compression of an existing surgical wound. Additionally, repositioning poses anesthetic challenges, including possible hemodynamic instability due to altered venous return and cardiac output, as well as respiratory compromise resulting from airway misalignment or accidental extubation.

## 2. Materials and Methods

A total of 13 cat cadavers and 9 dog cadavers (obtained from the Department of Pathology, University of Agricultural Sciences and Veterinary Medicine Cluj-Napoca, Romania, with the consent of their owners), varying in age, sex, size, and breed, were included in the study and divided into two distinct groups. Only animals without severe prior abdominal pathologies were included. Cadavers showing extensive adhesions preventing safe mobilization of the greater omentum were excluded from the study, such as ovariohysterectomy (OVH) or surgeries involving the urinary or gastrointestinal tract. Cadavers presenting minor adhesions of the greater omentum to the ventral abdominal wall caused by OVH were included, as these could be released by careful adhesiolysis.

In group A, the greater omentum was approached through a left lateral approach (LLA) laparotomy with the cadaver positioned in right lateral recumbency (RLR; Table 1). In group B, the omentum was accessed through a right lateral approach (RLA) laparotomy with the cadaver positioned in left lateral recumbency (LLR; Table 2).

**Figure 1:**
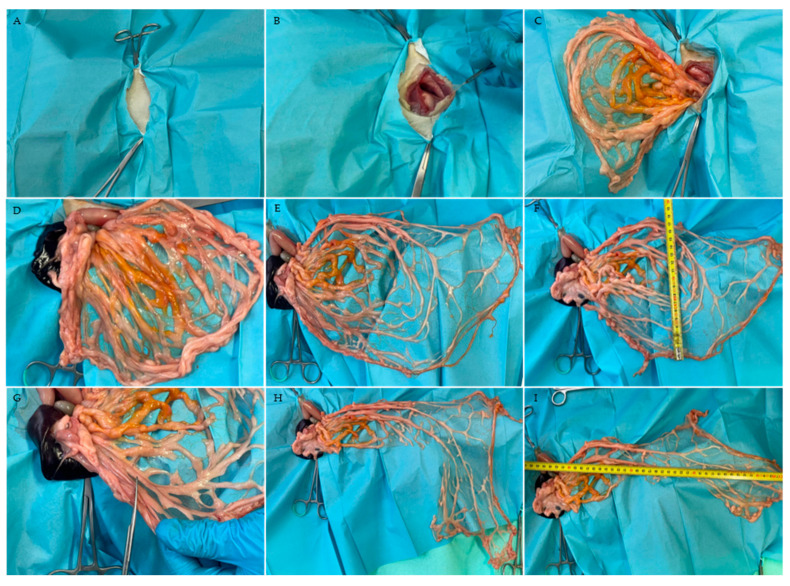
Cat in RLR with LLA. (**A**) Preparation of the incision site. (**B**) Cutaneous incision caudal to the last rib. (**C**) Gradual exposure of the greater omentum by carefully pulling the bursal portion in a caudal direction. (**D**) The double-folded bursal portion of the greater omentum pulled in a cranial direction. (**E**) Dorsal extension of the deep leaf. (**F**) Measurement of the width after dorsal extension (measured width: 17 cm). (**G**,**H**) Preparation and measurement of the inverted L-shaped pedicle flap. (**I**) Measurement of the length after inverted L-shaped omental pedicle elongation (measured length: 41 cm).

First, the 13th rib was identified, and the area caudal to it was clipped to prepare the cadaver for a lateral approach laparotomy. The incision was made approximately 3 cm caudal to the last rib and measured about 3–5 cm in length, depending on the size of the cadaver (Figure 1A).

After the cutaneous incision, the *M. obliquus externus abdominis*, identifiable by its caudoventrally oriented fibers, was bluntly separated. The underlying *M. obliquus internus abdominis* can be distinguished by its cranioventrally oriented fibers. The final muscular layer before reaching the peritoneum, the *M. transversus abdominis*, whose fibers run perpendicular to the spine, was carefully elevated with forceps to avoid damage to the abdominal organs while incising the muscle and peritoneum, which can easily be compromised when opening the abdomen in lateral recumbency (Figure 1B).

To maintain a clear surgical field and adequate visualization within the abdominal cavity, Gelpi or Farabeuf retractors were used.

For this study, the lengthening technique described by Ross and Pardo was applied. This technique includes dorsal extension of the deep leaf of the bursal portion followed by preparation of an inverted L-shaped omental pedicle flap. To prevent tissue dehydration, gauze soaked in 0.9% NaCl solution (B. Braun, Melsungen, Germany) was placed on the greater omentum to maintain tissue pliability during flap preparation.

In the cohort positioned in RLR with LLA laparotomy, the spleen was first exteriorized to identify the splenic and bursal portions of the greater omentum (Figure 1D). The largest component, the bursal portion, was carefully mobilized from its physiological position covering the intestinal coils and pulled caudally to its most caudal extent near the urinary bladder.

Subsequently, dorsal extension of the bursal portion was performed by incising the deep leaf close to the splenic artery and separating it from its attachments to the pancreas and dorsal abdominal wall (Figure 1E). In all cadavers, measurements of the length and width were obtained before and after dorsal extension (Figure 1F).

To allow extension of the omentum toward the limbs as well as the cervical region, an inverted L-shaped incision was created. The bursal portion was first mobilized by releasing its splenic attachments while preserving the gastroepiploic arcade. A transverse incision was then performed approximately to the mid-portion of the expanded bursal portion, taking into account the individual vascular pattern. Subsequently, a caudally directed longitudinal incision encompassing approximately two-thirds of the dorsally extended bursal portion was performed (Figure 1G). During preparation of the inverted L-shaped omental pedicle flap, care was taken to preserve a sufficient number of blood vessels to maintain perfusion to the most caudal part of the pedicle flap (Figure 1H,I).

**Figure 2:**
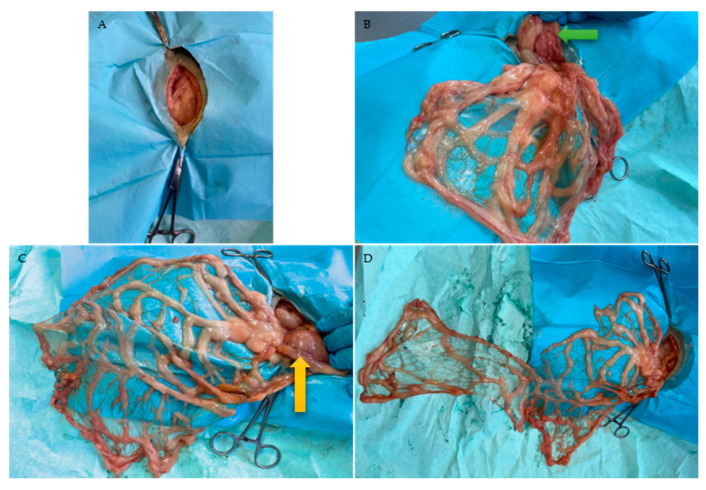
Dog in LLR with RLA. (**A**) Incision. (**B**) Exposure of the bursal portion of the greater omentum by gently pulling it in a caudal direction. The green arrow indicates the right pancreatic lobe adjacent to the proximal duodenum. (**C**) Dorsal extension of the greater omentum. The yellow arrow indicates the stomach and its gastroepiploic arcade. (**D**) Inverted L-shaped elongation.

**Figure 3:**
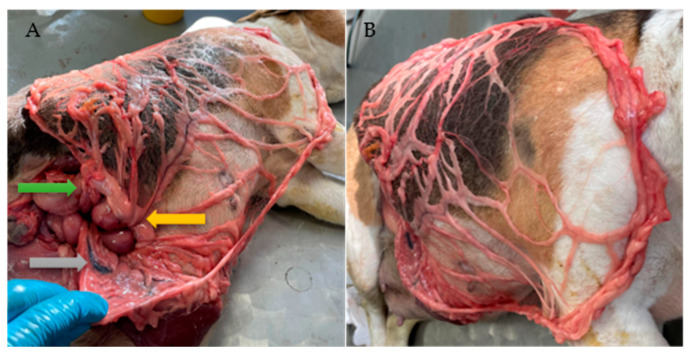
Dorsal extension technique in a Beagle (Group 2; RLA/LLR). (**A**) The gray arrow indicates the splenic artery. The yellow arrow indicates the origin of the omental branches from the gastroepiploic arcade, partially covered by adipose tissue. The green arrow indicates the proximal duodenum adjacent to the right pancreatic lobe. (**B**) The extended bursal leaf covers the thoracic region, reaching the cranial aspect of the shoulder joint.

In the second cohort, cadavers were positioned in LLR, and the abdominal cavity was entered through an RLA laparotomy (Figure 2). Depending on the degree of intestinal distension, the jejunum may enter the surgical field and must be carefully retracted, as well as the caudate process of the liver. To correctly identify the bursal portion of the greater omentum, the pylorus and proximal duodenum were used as anatomical landmarks. Mediodorsally, the right pancreatic lobe is attached to the proximal duodenum (Figure 2B and Figure 3A).

After identification of the bursal portion of the greater omentum, the deep leaf was carefully detached in a transverse direction from the dorsal abdominal wall and from the left lobe of the pancreas until the splenic artery was reached. The principle of dorsal extension was therefore identical to that performed in the first cohort but mirrored from the right to the left side. The elongated L-shaped omental pedicle flap was then created as described for the first group (Figure 2D).

In both groups, measurements of the bursal portion of the greater omentum were obtained before and after dorsal extension, as well as after preparation of the elongated L-shaped pedicle flap (Figure 1F,I). For all measurements, the starting point was defined as the attachment of the superficial leaf of the bursal portion at the greater curvature of the stomach, located approximately 0.5–1 cm below the gastroepiploic artery. Dimensions were obtained using a flexible measuring tape. The endpoint for the baseline measurement was defined as the most caudal point of the bursal portion, corresponding to the junction between the superficial and deep leaves. After dorsal extension and inverted L-shaped preparation, the most distant point from the greater curvature of the stomach was recorded.

The primary measurement was defined as the baseline value (100%). Relative length was calculated for both extension techniques as (modified length/baseline length) × 100. Subsequently, mean relative values and standard deviations were calculated for each technique (Table 3). This standardized calculation allowed comparison of the achieved omental elongation between cats, small dogs, and large-breed dogs (Table 1 and Table 2).

To test for differences between LLA and RLA laparotomies, two-tailed Mann–Whitney U tests were applied on the respective percentage values, with a significance level of α = 0.05. Analyses were performed using R (version 4.5.3).

## 3. Results

The LLA laparotomy performed in group A allowed evaluation of both the size and integrity of the bursal and splenic portions of the greater omentum in close proximity. In most cases, the spleen was the first organ identified during LLA laparotomy and, after gentle exteriorization, served as an anatomical landmark, as the splenic artery delineated the bursal and splenic portions of the greater omentum.

In all included cadavers, dorsal extension of the greater omentum could be performed without macroscopic impairment of tissue integrity (Figure 1E, Figure 2C and Figure 3A,B). However, further elongation through preparation of an elongated L-shaped omental pedicle flap resulted in minor structural damage in one dog (Dachshund, No. 11) and one cat (No. 2), whereas complete rupture of the pedicle flap occurred in cat No. 6.

In the second group (group B), with cadavers positioned in LLR, the bursal portion of the greater omentum was initially identified by its superficial leaf, connecting to the pylorus and the proximal duodenum. From there, it coursed dorsally and continued as the deep leaf, attaching to the dorsal abdominal wall and extending toward the left lobe of the pancreas before merging with the deep leaf of the splenic portion.

As in group A, dorsal extension of the greater omentum could be performed successfully in all cadavers. Further elongation of the bursal portion into an elongated L-shaped pedicle flap was successful in eight specimens, whereas partial structural damage occurred in cat No. 5 and complete rupture of the pedicle flap in cat No. 6.

No significant differences were found between the two groups for any of the evaluated parameters (all *p* > 0.05). After dorsal extension, both length and width remained similar (DE length: *p* = 0.224, U = 42.00; DE width: *p* = 0.131, U = 37.50). Comparable results were obtained following the preparation of the elongated L-shaped omental pedicle flap (IL length: *p* = 0.773, U = 33.00; IL width: *p* = 0.923, U = 35.00).

## 4. Discussion

In this study, the feasibility of accessing the greater omentum through both left and right lateral approach laparotomies was evaluated to provide practitioners with practical guidance for preparing an omental pedicle flap via a lateral approach. Both LLA and RLA laparotomies provided satisfactory visualization of the greater omentum. However, anatomical particularities related to body size and proportions in certain dog breeds may hinder access to the anchoring of the greater omentum at the greater curvature of the stomach. This was particularly evident in the Dachshund included in this study, in which the spleen and stomach were located unusually cranial, extending behind the eighth and ninth ribs. In this case, an extension of the incision by approximately 2 cm was required, together with the assistance of retractors to maintain adequate exposure of the surgical field.

In contrast, a classical midline laparotomy allows comfortable evaluation of the organs and structures within the abdominal cavity. The greater omentum is often the first structure observed, as it typically lies over the intestinal coils. Furthermore, identification of its anatomical attachments is easier, and dorsal extension of the deep leaf of the bursal portion can usually be performed without major impediments.

Both LLA and RLA laparotomies may present challenges for surgeons unfamiliar with this approach. Before performing dorsal extension, it is essential to identify the anatomical landmarks that allow correct identification of the bursal portion of the greater omentum.

During LLA laparotomy, the first structures encountered are typically the jejunum and the greater omentum covering the intestinal loops, as well as the left side of the stomach, including the fundus and body. Identification of the splenic hilum and splenic artery provides a reliable landmark separating the splenic and bursal portions of the greater omentum. The left epiploic artery originates from the splenic artery and runs within the deep leaf of the bursal portion of the greater omentum [13].

The bursal portion can then be carefully mobilized from its physiological position covering the intestinal coils.

When performing an RLA laparotomy, the pylorus and proximal duodenum serve as the principal landmarks for identifying the superficial leaf of the bursal portion, which then turns dorsally to continue as the deep leaf along the right side of the stomach. The left part of the bursal portion can be gently mobilized toward the incision site, taking care to avoid damage to the delicate omental tissue or its connections to the spleen. The deep leaf, with its attachments to the dorsal abdominal wall and the right pancreatic lobe, can then be gradually dissected using Metzenbaum scissors.

The vascularization of the right side of the greater omentum can be followed from the hepatic artery, which branches into the gastroduodenal artery and subsequently divides into the pancreaticoduodenal artery and the right gastroepiploic artery [16,17].

Stepwise dissection is essential during this procedure to avoid damage to the right pancreatic lobe, as the omental tissue transitions gradually into pancreatic tissue. Furthermore, progressive dissection toward the left side of the greater omentum is required to prevent injury to the splenic artery. Although the physiological position of the spleen and the fragile nature of the omental tissue may complicate the procedure, careful and gradual dissection allowed successful completion of dorsal extension in all cadavers included in the study.

In all specimens, dorsal extension could be achieved without major structural damage and almost doubled the surface area of the omentum. As the greater omentum in dogs is generally larger than in cats, dorsal extension alone may be sufficient in canine patients to reach the target surgical region [1]. However, the collected data suggest that this anatomical difference is not always reflected by the relative elongation achieved (Table 3). In one cat from group A, the relative length was more than tripled (312.5%), compared with a group mean of 203.1%. This specimen also showed a noticeable increase in omental width (12 cm to 20 cm), suggesting greater tissue mobility than observed in the remaining subjects. In the remaining cadavers, the omental width showed only minor changes after dorsal extension. This limited increase is reflected in the mean baseline percentages presented in Table 3.

Further elongation through an inverted L-shaped incision of the dorsally extended bursal portion was achieved without rupture in 17 of 22 cadavers (77.3%). In three specimens, partial tearing of the omental tissue occurred, whereas complete rupture of the pedicle flap was observed in two specimens.

The relative baseline percentages after inverted L-shaped preparation differed not only between cats and dogs but also among individuals of the same species. This variability is reflected by the standard deviation, indicating interindividual differences after dorsal extension and inverted L-shaped elongation. Such variability may be attributed to differences in the number and distribution of epiploic vessels, which may allow a wider transverse incision through the bursal portion of the greater omentum.

The two-tailed Mann–Whitney U test revealed no significant differences between the two groups for either technique (all *p* > 0.05). Hence, these findings suggest that both approaches, LLA and RLA laparotomies, provide comparable outcomes for dorsal extension as well as L-shaped elongation of the bursal portion. This allows the surgeon to choose the approach based on the target region and direction of omental transposition.

This technique was first described by Ross and Pardo (1993), who reported that the inverted L-shaped flap could potentially reach almost any region of the body, although limitations were expected in obese dogs or in animals with previous surgical interventions leading to adhesions [2].

A later study by Karl and Dupré (2012) investigated this technique in cats and reported that satisfactory elongation was achieved in only 50% of cases and adequate wound coverage in only half of those [18]. This reduced success rate may be related to the delicate structure of the feline greater omentum, which increases the risk of tissue rupture during elongation [18].

The greater omentum is often referred to as the “surgeon’s best friend” or the “policeman of the abdomen” due to its ability to adhere to tissues affected by infection or trauma [3,19].

However, adhesions of the greater omentum to adjacent structures due to previous surgical interventions may represent a significant obstacle for surgeons. In the present study, animals with severe adhesions resulting from previous urinary bladder or gastrointestinal surgeries were excluded.

One excluded cadaver, a Jack Russell Terrier, had previously undergone a caudal midline celiotomy. Examination through the lateral approach revealed a ruptured peritoneum, severe damage to the linea alba, and retained suture material. These defects were encapsulated by the caudal portion of the bursal part of the greater omentum.

Four additional cadavers showed severe adhesions of the greater omentum associated with previous surgical interventions, including apparent enterotomies in three animals and cystotomy in one.

These findings support the observations of Ross and Pardo that adhesions can hinder successful dorsal extension of the greater omentum, and they confirm the observations of Karl and Dupré that inverted L-shaped elongation is particularly challenging in feline patients due to the delicate structure of the omental tissue [2,18].

Adhesions caused by previous surgical interventions may lead to serious complications, including intestinal obstruction, strangulation of the small intestine due to omental entrapment, or complications related to gastropexy procedures [20,21].

Previous studies have also reported adhesions of the genital tract to intra-abdominal organs or the abdominal wall, as well as granuloma formation resulting from inadequate antiseptic procedures or retained ovarian tissue [22,23].

In horses undergoing abdominal surgery, adhesions were reported in approximately 15% of cases, partly due to the lack of preventive protocols, such as minimizing surgical manipulation, achieving thorough hemostasis, and performing adequate lavage [24]. Postoperative adhesions in small animals may remain undetected due to shorter lifespans, limited diagnostic investigations, and the relatively infrequent use of necropsy examinations [20]. This assumption is supported by the findings of the present study, in which five cadavers were excluded due to severe adhesions of the greater omentum. Furthermore, the angiogenic potential of the greater omentum has been demonstrated in several studies, with newly formed blood vessels frequently observed in tissues to which the omentum adheres [25,26].

Understanding the vascularization of the greater omentum is crucial for successful preparation of an omental pedicle flap, particularly the epiploic vessels originating from the gastroepiploic arcade [2,14,27].

In contrast to humans, dogs and cats lack a middle epiploic artery [2]. Nevertheless, the vascular pattern of the greater omentum may vary considerably among individuals. Variations have been reported in the branching patterns of the celiac artery and its branches, including the hepatic, splenic, and left gastric arteries. In some cases, the celiac artery may arise as a common trunk together with the cranial mesenteric artery [28,29].

Because both adhesions and vascular anatomy influence the success of omental transposition, preoperative diagnostic evaluation would be desirable, although this is not always feasible. In human medicine, several protocols have been developed to assess peritoneal adhesions preoperatively. Dynamic ultrasonography has been used to assess the “visceral slide” phenomenon, which describes the normal gliding movement of abdominal organs along the inner abdominal wall during respiration and can help detect intra-abdominal adhesions [30,31].

However, data in veterinary medicine remain limited. One study involving 88 dogs and 17 cats reported that adhesions or metastases within the abdominal cavity are often difficult or impossible to detect using ultrasonography [32]. Furthermore, the diagnostic value of ultrasonography may depend strongly on the experience of the clinician performing the examination [33,34].

Therefore, consideration of species, breed, age, and complete patient history remains essential when assessing the potential risk of intra-abdominal adhesions.

The main limitation of the present study is the relatively small number of cadavers and the use of cadaveric specimens. Also, the vitality and viability of the omental pedicle flap in both techniques could not be evaluated due to the cadaveric nature of the study. However, recent clinical studies suggest that vascularization of the omental pedicle flap in extra-abdominal procedures can be successfully monitored by visualization of the blood flow using Doppler ultrasonography [35,36]. Future studies should investigate the development of diagnostic protocols using ultrasonography or other imaging modalities to identify adhesions prior to omental transposition. Additionally, laparoscopic-assisted transposition of the greater omentum may represent a promising future approach, allowing the surgeon to inspect the abdominal cavity visually while facilitating preparation of an omental pedicle flap using minimally invasive techniques.

## 5. Conclusions

Performing an omentopexy has proven to be an effective adjunctive maneuver for various pathologies. Surgeons in both veterinary and human medicine appreciate this versatile organ and its beneficial properties. When the surgical target lies within the abdominal cavity, a standard midline laparotomy is usually sufficient. However, in cases involving pathologies affecting the limbs, thoracic cavity, or soft tissues of the neck, a lateral approach laparotomy may be more effective and safer, as repositioning the patient carries both anesthesiologic and surgical risks. Both right and left lateral approach laparotomies present certain challenges, including the topographical position of internal organs and the greater omentum. The preferred technique involves either dorsal extension of the bursal portion of the greater omentum or further elongation by creating an inverted L-shaped pedicle flap. Both techniques have been previously described and, when combined with a lateral approach laparotomy, may provide a promising alternative for clinicians seeking access to the greater omentum. The absence of significant differences between LLA and RLA indicates that both approaches may be considered equally suitable for clinical application. However, adhesions of the greater omentum to the abdominal wall resulting from previous surgical interventions may represent a significant impediment to the creation of a viable omental pedicle flap and, depending on their severity, may even make its preparation impossible. Future studies should therefore focus on methods for detecting adhesions prior to surgery.

## Figures and Tables

**Table 1:** Group A: Experimental group using LLA with RLR.

No.	Species	Age Group	Gender	Breed	Weight (kg)	O Length	O Width	DE Length	DE Width	IL Length	IL Width	Rupture/Damage
1	Cat	Adult	F	ESH	2.6	8.0 *	12.0 *	25.0 (312.5%)	20.0 (166.7%)	39.0 (487.5%)	10.0 (83.3%)	-
2	Cat	Adult	M	ESH	2.9	15.0 *	20.0 *	34.0 (226.7%)	22.0 (110.0%)	-	-	Damage
3	Cat	Juvenile	F	ESH	1.4	14.0 *	13.5 *	24.0 (171.4%)	14.0 (103.7%)	36.0 (257.1%)	7.0 (51.9%)	-
4	Cat	Adult	M	ESH	3.5	14.0 *	16.0 *	31.0 (221.4%)	19.0 (118.8%)	54.0 (385.7%)	12.0 (75.0%)	-
5	Cat	Adult	M	ESH	3.7	15.5 *	17.5 *	32.5 (209.7%)	21.5 (122.9%)	43.0 (277.4%)	10.0 (57.1%)	-
6	Cat	Adult	M	ESH	3.1	12.5 *	12.0 *	29.0 (232.0%)	14.0 (116.7%)	-	-	Rupture
7	Cat	Adult	M	ESH	4.4	14.5	15.0	26.0 (179.3%)	17.0 (113.3%)	41.0 (282.8%)	9.0 (60.0%)	-
8	Dog	Adult	M	Golden Retriever	30.5	35.0 *	34.0 *	61.0 (174.3%)	27.0 (79.4%)	79.0 (225.7%)	12.5 (36.8%)	-
9	Dog	Adult	M	German Sheperd	32.4	32.5 *	27.0 *	66.5 (204.6%)	29.0 (107.4%)	86.0 (264.6%)	25.5 (94.4%)	-
10	Dog	Juvenile	F	Mixed-breed	5.7	15.0 *	14.5 *	23.5 (156.7%)	14.5 (100.0%)	35.0 (233.3%)	8.0 (55.2%)	-
11	Dog	Adult	F	Dachshund	9.0	20.0 *	18.5 *	31.0 (155.0%)	18.0 (97.3%)	-	-	Damage

Abbreviations: ESH—European Shorthair; M—Male; F—Female; O—omentum; DE—dorsal extension; IL—inverted L shape. Values in cm; * = baseline value (100%); values in parentheses represent percentage of baseline length and width.

**Table 2:** Group B: Experimental group using RLA with LLR.

No.	Species	Age Group	Gender	Breed	Weight (kg)	O Length	O Width	DE Length	DE Width	IL Length	IL Width	Rupture/Damage
1	Cat	Adult	F	ESH	2.3	13.0 *	14.0 *	30.0 (230.8%)	14.0 (100.0%)	52.0 (400.0%)	7.5 (53.6%)	-
2	Cat	Adult	M	ESH	3.2	17.5 *	14.5 *	34.5 (197.1%)	20.5 (141.4%)	54.0 (308.6%)	10.0 (69.0%)	-
3	Cat	Adult	F	ESH	3.9	16.0 *	12.5 *	27.0 (168.6%)	20.0 (160.0%)	39.0 (243.8%)	8.0 (64.0%)	-
4	Cat	Adult	M	ESH	3.7	15.0 *	18.0 *	29.0 (193.3%)	24.0 (160.0%)	44.0 (293.3%)	10.0 (55.6%)	-
5	Cat	Juvenile	M	ESH	1.7	11.0 *	12.0 *	22.0 (200.0%)	14.0 (116.7%)	-	-	Damage
6	Cat	Adult	F	ESH	3.4	20.0 *	14.0 *	33.0 (165.0%)	20.0 (142.9%)	-	-	Rupture
7	Dog	Adult	F	Mixed-breed	19.4	15.0 *	11.0 *	27.0 (180.0%)	11.0 (100%)	41.0 (273.3%)	7.0 (63.6%)	-
8	Dog	Adult	M	Mixed-breed	8.6	18.0 *	15.0 *	27.0 (150.0%)	18.0 (120.0%)	36.0 (200.0%)	11.0 (73.3%)	-
9	Dog	Adult	F	Beagle	15.3	16.0 *	24.0 *	34.0 (212.5%)	32.0 (133.3%)	52.0 (325.0%)	15.5 (64.6%)	-
10	Dog	Adult	M	Cane Corso	38.1	38.0 *	32.0 *	62.0 (163.2%)	34.0 (106.3%)	72.0 (189.5%)	17.0 (53.1%)	-
11	Dog	Adult	F	Pumi Dog	10.2	18.0	17.0	25.5 (138.9%)	20.0 (117.7%)	45.0 (250.0%)	11.0 (64.7%)	-

Abbreviations: ESH—European Shorthair; M—Male; F—Female; O—omentum; DE—dorsal extension; IL—inverted L shape. Values in cm; * = baseline value (100%); values in parentheses represent percentage of baseline length and width.

**Table 3:** Relative omental length and width expressed as mean percentage of baseline with standard deviation (mean ± SD).

	DE Length (%)	DE Width (%)	IL Length (%)	IL Width (%)
**Group A**	203.1 ± 45.8%	112.4 ± 22.4%	301.8 ± 86.9% *	64.2 ± 18.5% *
**Group B**	181.8 ± 26.9%	127.1 ± 21.6%	276.0 ± 68.4% **	62.4 ± 7.4% **

Abbreviations: DE—dorsal extension; IL—inverted L shape; SD—standard deviation; * = 8 specimens; ** = 9 specimens.

## Data Availability

The data presented in this study are available within the article.
